# Suicidal ideation, self-injury, aggressive behavior and substance use during intensive trauma-focused treatment with exposure-based components in adolescent and young adult PTSD patients

**DOI:** 10.1186/s40479-021-00172-8

**Published:** 2022-01-03

**Authors:** Anne Fischer, Rita Rosner, Babette Renneberg, Regina Steil

**Affiliations:** 1 Department of Clinical Psychology and Psychotherapy, Institute of Psychology, Goethe University Frankfurt, Varrentrappstr. 40-42, 60486 Frankfurt am Main, Germany; 2 Department of Psychology, Catholic University Eichstätt-Ingolstadt, Ostenstr. 25, 85072 Eichstätt, Germany; 3 Clinical Psychology and Psychotherapy, Freie Universität Berlin, Habelschwerdter Allee 45, 14195 Berlin, Germany

**Keywords:** Child abuse, Adolescents, Self-injury, Aggression, Substance use, Suicidality, Daily diary, Exposure

## Abstract

**Background:**

Multiple traumata such as child sexual and/or physical abuse often result in complex psychopathologies and a range of associated dysfunctional behaviors. Although evidence-based interventions exist, some therapists are concerned that trauma-focused psychotherapy with exposure-based elements may lead to the deterioration of associated dysfunctional behaviors in adolescents and young adults. Therefore, we examined the course of suicidal ideation, self-injury, aggressive behavior and substance use in a group of abuse-related posttraumatic stress disorder (PTSD) patients during phase-based, trauma-focused PTSD treatment.

**Methods:**

Daily assessments from a randomized controlled trial (RCT) of Developmentally adapted Cognitive Processing Therapy (D-CPT) were analyzed to test for differences in the stated dysfunctional behaviors between the four treatment phases. We conducted multilevel modeling and repeated measure ANOVAs.

**Results:**

We did not find any significant differences between the treatment phases concerning the stated dysfunctional behaviors, either at the level of urge or at the level of actual actions. On the contrary, in some primary outcomes (self-injury, aggressive behavior), as well as secondary outcomes (distress caused by trauma, joy), we observed significant improvements.

**Discussion:**

Overall, during D-CPT, adolescents and young adults showed no deterioration in dysfunctional behaviors, while even showing improvements in some, suggesting that trauma-focused treatment preceded by skills building was not deleterious to this population. Hence, the dissemination of effective interventions such as D-CPT should be fostered, whilst the concerns of the therapists regarding exposure-based components need to be addressed during appropriate training. Nevertheless, further studies with momentary assessment, extended measurement methods, a control group and larger sample sizes are needed to confirm our preliminary findings.

**Trial registration:**

The trial was registered at the German Clinical Trial Registry (GCTR), DRKS00004787, 18 March 2013, https://www.drks.de/DRKS00004787.

## Background

Child sexual and/or physical abuse (CA) is associated with a variety of mental health consequences and impairments that can last into adulthood [1–3]. In youth, the risk for a posttraumatic stress disorder (PTSD) is especially high after such an interpersonal traumatization [4]. Furthermore, adolescent PTSD patients often show emotion regulation difficulties [5] and consequently are likely to engage in high-risk problem behavior such as self-injury, substance use or suicidal ideation [3, 6, 7]. In comparison to PTSD after a single trauma, PTSD after long CA is more frequently characterized by these comorbid problems and a more severe psychopathology [8, 9]. Therefore, the recently released ICD-11 [10] comprises the new diagnosis termed “complex PTSD” (CPTSD). In addition to the core PTSD symptom clusters, CPTSD also includes symptoms of disturbed self-organization such as interpersonal problems, emotion regulation difficulties and negative self-concept [10].

Over the past few years, a variety of trauma-focused treatments for adolescents and young adults with PTSD have been developed for which meta-analyses reported overall medium to large effect sizes [11, 12]. Treatment success has been shown to be stable in the long-term [13]. Since controlled treatment studies solely focusing on PTSD after CA are sparse, Rosner et al. [14] conducted a randomized controlled trial (RCT) examining Developmentally adapted Cognitive Processing Therapy [D-CPT; 15] agains t a wait-list condition with treatment advice (WL/TA) in an adolescent and young adult sample with abuse-related PTSD. They found large effects on blind-rated PTSD symptom severity as well as comorbid sy mptoms (e.g., depression, borderline symptom severity). 

Despite substantial evidence for the effectiveness of trauma-focused psychotherapy for treating PTSD [11, 12], there is an insufficient dissemination in routine care [16, 17]. If traumatized youths receive no treatment or only unspecific, non-evidence-based interventions, this often leads to chronic impairments and poor results in adulthood [1, 18]. Cited reasons for an inadequate treatment supply are for example negative beliefs of therapists towards exposure elements [19–22]. In particular, trauma-focused treatment requires exposure with trauma-related experiences [23], although studies among clinicians revealed that exposure is an underutilized approach [19, 22], and rarely endorsed by child psychotherapists [24]. Clinicians have stated to feel uncomfortable in directly addressing traumatic experiences because they are concerned that patients experience too much distress during this exposure [19, 25–27]. The individual extent of these negative beliefs has been associated with the therapist’s qualifications (e.g., level of trauma expertise) [19, 22], the outlined underuse of exposure [21, 22, 24, 28–30] and its suboptimal delivery [20, 24, 26, 30].

In particular, the idea that exposure might lead to a deterioration in psychopathological symptoms is widely common [19, 22, 27, 29]. The therapists’ fear of symptom deterioration applies especially to PTSD patients with comorbid conditions [19, 22] and refers not only to actual PTSD symptoms but also to associated problems [22]. As a consequence, trauma-focused treatments with exposure elements are rated to be less appropriate if the patient is a victim of multiple CA [22]. This is in line with our vast experience in disseminating empirically based treatments for PTSD in adolescents and (young) adulthood. It shows that therapists often do not provide trauma-focused treatment with exposure elements because they fear that exposure elements are too distressing and elevate the risk for patients in engaging in suicidal, self-injurious, aggressive or substance use behavior. As D-CPT for abuse-related PTSD includes generating a trauma-narrative [15], therapists’ concerns regarding exposure may also hamper the dissemination of this approach.

Research findings on adults with PTSD indicate that the therapists’ fears of symptom deterioration are not in line with empirical findings [23, 31, 32]. Larsen et al. [31] focused on symptom deterioration during treatment, analyzing data from RCTs with victims of interpersonal violence*,* comparing CPT, prolonged exposure (PE) and CPT without exposure elements. No significant differences between the treatment approaches were observed and even those few patients who showed temporary deterioration experienced a significant improvement in PTSD severity at post-treatment [31]. Similar results have also been found for associated dysfunctional behaviors and comorbid conditions. Van Minnen et al. [33] examined secondary outcomes of 18 RCTs on PE. They analyzed if common comorbid conditions (e.g., substance use disorders) or associated problems (e.g., suicidality) worsen at the end of therapy. Their findings showed that not only PTSD symptoms but also comorbidities and associated problems were reduced. Referring to adolescents and young adults, single RCTs of PTSD therapy with exposure elements, as well as interventions with prefaced skill-building, revealed that secondary outcome-measures, such as depression, emotion regulation difficulties, interpersonal problems or suicidality, also improved after treatment [e.g. 34, 35]. Nevertheless, a need for further studies exists focusing explicitly on adolescents and young adults as well as data on dysfunctional behaviors during the course of trauma-focused treatment with exposure elements.

In this context, it is essential to understand the connection between dysfunctional behaviors and PTSD in adolescents and young adults. In a systematic review, Panagioti et al. [36] found a significant association between suicidality and PTSD for several adolescent trauma samples. However, they noted that the studies available do not provide sufficient evidence to explain possible underlying mechanisms of this association. There are several explanatory models discussed; for example, it is postulated that certain PTSD clusters such as re-experiencing or avoidance elevate the risk for suicide attempts [37]. Other researchers have suggested that affective or cognitive processes, such as the perception of hopelessness [38], serve as mediators. In fact, it has been shown that trauma-focused treatment can reduce suicide ideation in adults [39, 40].

In addition to suicidal ideation, CA also elevates the risk for engaging in self-injurious behavior [2, 41]. Deliberate injuries without suicidal intentions are also referred to as non-suicidal self-injury [42]. In this context, PTSD symptomatology is considered a potential mediator for the association between CA and self-injuries [41, 43]; for instance, self-injury may serve as a dysfunctional coping strategy against burdening re-experiences [43, 44]. In adult samples, trauma-focused psychotherapy has resulted in a reduction of self-injuries [45–47].

Furthermore, traumatic events and PTSD symptoms are connected with aggressive behavior [48–50], especially in patients with CPTSD [51]. Although most findings refer to adults, CA and PTSD are also strongly associated with violent behaviors [52] and aggression [53, 54] in adolescents; PTSD symptoms, again, have been discussed to mediate this relationship [52, 55]. Nevertheless, PTSD symptoms cannot fully explain the association between a trauma and physical aggression in adolescents and young adults [52]. Research with veterans indicated that the ability to regulate emotions is determining in whether PTSD patients exhibit impulsive aggressive behaviors [56]. For adolescents and young adults, it has also been recommended to promote emotion regulation skills during treatment in order to reduce violent behaviors [52].

The association between PTSD and an increased substance use is well documented in literature for adults [57, 58], but there is also growing evidence for this association in adolescent populations [59, 60]. Once again, a mediating role of avoidance symptomatology is suspected in which increased substance use is often described as self-medication or a dysfunctional coping mechanism following CA in PTSD patients [e.g., 61]. Findings from adults indicate that treatment improvements in PTSD are connected with subsequent improvements in substance use [33].

As stated above, against the concerns of some therapists, existing evidence indicates that treatments with exposure elements do not deteriorate PTSD severity or dysfunctional behaviors [31–33]. Nevertheless, there is still a need for studies focusing explicitly on adolescents and young adults with abuse-related PTSD and severe psychopathologies [15, 62]. Consequently, data on associated dysfunctional behaviors such as suicidal ideation, self-injury, aggressive behavior and substance use is required. In the RCT on D-CPT [14] daily assessments (diary cards) were used to monitor dysfunctional behaviors, providing the opportunity to determine if adolescents and young adults who have been victims of CA show deteriorations in these problem behaviors during D-CPT. In this study, we differentiate between the urge to engage in these dysfunctional behaviors and the actions that actually take place, while addressing the following research questions:

1. Are there any differences between the D-CPT treatment phases regarding the urge to engage in suicidal, self-injurious, aggressive or substance use behavior?
2. Are there any differences between the D-CPT treatment phases regarding actions of self-injurious, aggressive or substance use behavior?

In addition, broader self-report items were assessed to reflect the impact of the interventions during D-CPT on the patients’ well-being. Therefore, on an explanatory level, we also examined the differences between the treatment phases in terms of self-reported distress caused by trauma and joy as secondary outcomes.

## Methods

### Procedure and participants

This is an analysis of the data collected from an RCT during D-CPT; more detailed information on the procedure can be retrieved from Rosner et al. [14]. Adolescents and young adults aged 14–21 years were enrolled between July 2013 and June 2015 in Frankfurt, Berlin and Eichstätt-Ingolstadt in Germany. The respective ethics committees of all participating universities approved the study. After giving informed consent (which, in the case of minors, was also obtained from their caregivers) and having completed the baseline assessment, participants were randomly allocated to either the D-CPT or the WL/TA groups. Daily assessments of the stated dysfunctional behaviors and the respective urges were collected from the patients in the D-CPT group via diary cards. The requirement for participation in this study was a primary CA-related PTSD diagnosis according to DSM-IV [63] with a lowered threshold for avoidance symptoms [64]. Furthermore, the subjects should not be in receipt of pharmacotherapy or be on stable medication (for ≥3 weeks). Participants were excluded if they had an IQ ≤ 75. Other exclusion criteria were life-threatening suicidality, self-injury or self-harming behavior within the last six months. In addition, pervasive developmental disorders, concurrent psychotherapy, a diagnosis of lifetime psychotic or bipolar disorders also led to exclusion. Adolescents or young adults with a substance-induced disorder or a current substance dependence (including abstinence < 6 months) were, likewise, not included.

### Treatment

Patients received D-CPT tailored to treat adolescents and young adults who have experienced CA [15]. A pilot D-CPT study showed that the intervention could be carried out safely [15]; the findings of the main trial [14] are in line with this. In addition, no suicide attempts were registered in the D-CPT group [14]. D-CPT consisted of four treatment phases: building commitment (5 sessions), emotion regulation training (6 sessions), intensive Cognitive Processing Therapy (CPT; 15 sessions) and working on developmental tasks (4 sessions). More information on the D-CPT phases and the underlying rationale can be found in Matulis et al. [15]. The intervention consisted of 30 sessions (50 min each) and six optional additional units for crisis intervention or joint sessions with the caregiver. The 14 licensed therapists or therapists in training, who were trained in a 3-day D-CPT workshop and a subsequent refreshing workshop, delivered the treatment. Moreover, biweekly case consultations via telephone were offered.

### Measures

#### Diary cards

Patients received diary cards to monitor their daily dysfunctional behaviors. One card displayed one week of the treatment period. We asked adolescents and young adults to complete all items on a daily basis at a regular time each day. Participants rated suicidal ideation as well as the secondary outcomes of distress caused by trauma and joy on a 6-point scale ranging from 0 (*none*) to 5 (*excessively strong*). For self-injury, aggressive behavior and substance use, we differentiated between the urge to engage in these behaviors and actual actions. Urge was rated on a scale from 0 (*none*) to 5 (*no longer controllable*). Actions were documented as *yes* or *no*. The last item was a free-text field where patients could document further non-specified behaviors classified as *others*. Such daily assessments are ecologically valid ways to gather long-term data via self-report and to minimize retrospective biases [65]. The repeated nature of the measurement allows the elucidation of the dynamics of psychopathological symptoms [66]. In terms of therapeutic benefit, the information provided was used for the identification and application of stress tolerance as well as emotion regulation strategies [15].

#### Clinician-administered PTSD scale for children and adolescents (CAPS-CA)

The CAPS-CA [67, 68] is a widely used structured clinical interview to assess PTSD according to DSM-IV [63] and was applied at the study intake. Trained, independent raters scored the severity of symptoms on a scale ranging from 0 (*never/no problem*) to 4 (*most of the time/extreme*), thereby, the maximum sum is 136, with higher scores indicating more severe symptoms. Cronbach’s alpha for the total sum score was *α* = .875. Subscales had acceptable to good reliability (intrusion *α* = .785; avoidance *α* = .626; hyperarousal *α* = .618).

#### University of California at Los Angeles PTSD Reaction Index for DSM-IV (Revision 1; UCLA-PTSD-RI)

The German version [69] of the UCLA-PTSD-RI [70] was applied to measure self-reported trauma exposure as well as PTSD symptoms. After a brief screening of lifetime trauma, the A1 and A2 criteria of DSM-IV PTSD were examined, followed by 22 items assessing the frequency of PTSD symptoms during the past month. Answers were given on a 5-point Likert Scale ranging from 0 (*none)* to 4 (*most of the time*). The maximum total sum score is 68. Again, higher scores indicated a greater symptom severity. In our sample, the sum score had a good reliability *⍺* = .841, whereas the internal consistencies for the subscales were acceptable (intrusion *⍺* = .785; avoidance *⍺* = .615; hyperarousal *⍺* = .627).

### Data analysis

Firstly, we assigned the observations of the diary cards to the treatment phases. For a descriptive overview, the mean values and standard deviations of each variable per phase were determined (Tables 2 and 3). The collected data comprised a nested structure, with repeated measures (Level 1) being clustered within patients (Level 2). Due to the nested data structure, a number of participant dropouts during the trial and the varying numbers of observations, we chose to analyze the differences in dysfunctional behaviors between the treatment phases at the level of urge (research question 1) with multilevel modeling (MLM) [66]. In comparison to classic repeated-measures analysis of variance (ANOVA), MLM is more flexible in dealing with missing data and every observation can be included in the estimation [71].

The models were set up gradually [72]. Firstly, individual differences were modeled at the beginning of the treatment in a random intercept model. If the interclass correlation (ICC) deviated significantly from 0, grouping effects were presumed, in this case, we proceeded within the MLM framework. Subsequently, the respective treatment phase was included as a fixed predictor for the dysfunctional behavior (e.g., for self-injury). In a model comparison, we tested whether to include a random slope for the treatment phase in the third step. Using the final model, we analyzed whether a patient’s urge for dysfunctional behavior differed between the respective phases; this was confirmed if there was a significant difference regarding the slope within a treatment phase compared to the slope at the beginning of treatment (phase 1). We allowed free covariation of the random effects; their significance was evaluated with likelihood ratio tests. The model fit was estimated by comparing the random variance to a model without random variance. By using a default estimation of degrees of freedom, significances for the fixed effects were calculated. Secondary outcomes (distress caused by trauma, joy) were analyzed analogously.

The differences in dysfunctional behaviors between the treatment phases at the level of actual actions were analyzed with repeated-measure ANOVAs (research question 2). Firstly, we computed the mean actions per patient for each phase. Subsequently, we conducted an ANOVA using this value as a dependent variable and the treatment phase as a within-subject factor. Mauchly’s test was used to check for sphericity [73].

For all tests, we applied a common alpha level of .05. Statistical analyses were proceeded with R-Studio and IBM SPSS Statistics 27 for Windows.

## Results

*N* = 44 patients were randomized to the D-CPT group. Table 1 contains the demographic information and baseline PTSD scores of the sample.

**Table 1:** Demographic information and baseline scores

	D-CPT Group (*n* = 44)
Age, mean (95% *CI*)	18.2 (17.5–18.8)
Female, No. (%)	39 (89)
Posttraumatic Stress Symptom Score	
CAPS-CA, *M (SD)*	65.61 (23.55)
UCLA-PTSD-RI, *M* (*SD*)	41.20 (10.75)
Comorbid DSM-IV disorders, No. (%)	
0	10 (23)
1 or 2	23 (52)
≥ 3	11 (25)
Trauma, No. (%)	
Physical only	11 (25)
Sexual only	7 (16)
Both	26 (59)
Former self-injury, No. (%)	32 (72.7)
Present self-injury, No. (%)	15 (34.1)
No. of suicide attempts before treatment, mean (95% CI)	0.7 (0.3–1.5)

Note: *CAPS-CA* Clinician-Administered PTSD Scale for Children and Adolescents for DSM-IV, *D-CPT* Developmentally adapted Cognitive Processing Therapy, *PTSD* posttraumatic stress disorder, *UCLA-PTSD-RI* University of California at Los Angeles Posttraumatic Stress Disorder Reaction Index, *CI* confidence interval, *M* mean, *SD* standard deviation

### Response rates and descriptive data on dysfunctional behaviors

The number of diary cards (*n*_*diar*y_) as well observations in terms of diary card entries (*n*_*obs*_.) that were taken into account for the calculations varied for each phase and each variable because of several aspects. Missing diary cards can be assigned to the following reasons: there were 7 patients from whom no diary cards could be included in the analyses (2 participants never started therapy for organizational reasons, 2 patients had been erroneously randomized, and 1 patient did not respond to contact attempts shortly after intake). Diary cards were also missing from 2 patients who had actually finished the therapy but whose diary cards were, unfortunately, not collected post-treatment. Consequently, diary cards were (at least partially) available from *n* = 37 patients. Within these, there were drop-outs during the course of treatment that likewise diminished the number diary cards (*n*_*diar*y_) and, consequently, the number of observations (*n*_*obs*_): 2 patients dropped out during phase 1, 5 patients during phase 2 and 3 patients during the third phase.

Secondly, the number of diary cards (*n*_*diar*y_) and observations (*n*_*obs.*_) per phase varied due to different lengths of the treatment phase (e.g., 15 sessions in treatment phase 3 vs. 4 sessions in treatment phase 4). Additionally, the phase duration varied between the patients as a consequence of the additional optional joker sessions for crisis intervention or joint sessions with the caregiver. The last reason for differences in available observation (*n*_*obs.*_) for each variable studied are missing entries on the diary cards. Although the therapists repeatedly asked them to monitor their daily dysfunctional behaviors, the compliance to answer the diary cards on a regular, daily basis varied between the participants.

All in all, *n*_*diary*_ = 4044 diary cards were included. To determine the response rates, we related the patient’s number of treatment days to the number of available diary cards. In this sample, the average response rate was *m*_*response*_ = 85.7%; only a few outliers had a rate below 40%. The mean response rates for the different treatment phases were *m*_*response*_ = 87.7% (phase 1), *m*_*response*_ = 88.4% (phase 2), *m*_*response*_ = 82.6 (phase 3) and *m*_*response*_ = 82.4 (phase 4). About 75% (*n*_*diary*_ = 2782) of the available diary cards were answered completely with *n*_*obs*_ = 45 each for the whole week; the remaining diary cards had at least one missing entry.

Table 2 includes all means and standard deviations concerning the urge to engage in dysfunctional behaviors as well as distress caused by trauma and joy. Table 3 presents the mean number of dysfunctional behaviors per treatment phase. Regarding the option *others*, patients entered further dysfunctional behaviors that were not displayed such as *dissociation*, *spending a lot of money*, *binge eating* or *vomiting*.

**Table 2:** Means of diary card items (urge) in different treatment phases and secondary outcomes

Treatment phase	Phase 1		Phase 2		Phase 3		Phase 4	
	*n*_*obs.*_	*M (SD)*	*n*_*obs.*_	*M (SD)*	*n*_*obs.*_	*M (SD)*	*n*_*obs.*_	*M (SD)*
Suicidal ideation	1267	0.46 (0.99)	936	0.45 (1.04)	1270	0.54 (1.26)	382	0.93 (1.51)
Self-injury	1286	0.57 (1.16)	929	0.50 (1.16)	1259	0.55 (1.30)	380	0.96 (1.55)
Aggressive behavior	1285	0.64 (1.15)	912	0.55 (1.07)	1224	0.47 (0.99)	340	0.45 (0.97)
Substance use	1278	0.31 (0.91)	917	0.27 (0.76)	1253	0.39 (1.07)	378	0.44 (1.12)
Distress caused by trauma	1291	2.25 (1.32)	943	2.10 (1.36)	1326	1.99 (1.41)	378	2.03 (1.14)
Joy	1324	1.94 (1.21)	943	2.10 (1.23)	1327	2.05 (1.31)	380	2.14 (1.13)

*Note*: *M* mean, *SD* standard deviation, *n*_*obs.*_ number of observations in terms of diary card entries. Differences in *n*_*obs.*_ Are due to missing data and different lengths of treatment phases. Scores range from 0 to 5 with higher scores indicating a greater urge to engage in the dysfunctional behaviors

**Table 3:** Means of diary card items (actions) in different treatment phases

Treatment phase	Phase 1		Phase 2		Phase 3		Phase 4	
	*n*_*obs.*_	*M (SD)*	*n*_*obs.*_	*M (SD)*	*n*_*obs.*_	*M (SD)*	*n*_*obs..*_	*M (SD)*
Self-injury	1008	0.06 (0.24)	793	0.05 (0.21)	1000	0.03 (0.17)	322	0.11 (0.31)
Aggressive behavior	1006	0.06 (0.24)	777	0.05 (0.21)	988	0.07 (0.25)	229	0.04 (0.19)
Substance use	1002	0.06 (0.24)	764	0.06 (0.23)	995	0.05 (0.22)	318	0.03 (0.18)

*Note: M* mean, *SD* standard deviation, *n*_*obs.*_ number of observations in terms of diary card entries. Differences in *n*_*obs.*_ Are due to missing data and different lengths of treatment phases. Scores range from 0 (no action) to 1 (action took place)

### Research question 1

**Fig. 1:**
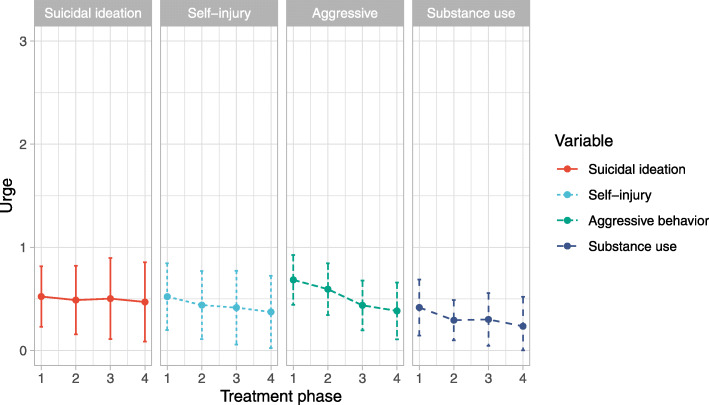
Course of the fixed effects from multilevel modeling for suicidal ideation, self-injury, aggressive behavior and substance use (urge) during D-CPT treatment phases. Scores range from 0 to 5 with higher scores indicating a greater urge to engage in the dysfunctional behaviors. Error bars represent the standard errors of the estimates

In order to test for differences between the treatment phases at the level of urge, we conducted MLM. As time was coded 0 for the first phase, the intercepts (fixed effects) provided an estimate of the baseline scores in phase 1. In the final random intercept with random slope model, phase-specific changes in comparison this baseline term were estimated for phase 2, phase 3 and phase 4 (Table 4). The random effects describe the approximated differences in phase 1 between patients (random intercept) as well as the patient-specific change for each phase (random slopes). Figure 1 shows the changes in effects from the multilevel modeling during the course of treatment.

**Table 4:** Fixed effects from multilevel modelling of diary card items (urge) as well as secondary outcomes

	*Estimate (SE)*	*t (DF)*	*p*
Suicidal ideation			
Intercept	0.53 (0.15)	3.49 (34.94)	<.000 ***
Phase 2	−0.03 (0.05)	− 0.71 (30.96)	.482
Phase 3	−0.02 (0.08)	− 0.25 (31.71)	.804
Phase 4	−0.05 (0.09)	− 0.59 (30.09)	.562
Self-injury			
Intercept	0.52 (0.16)	3.17 (36.17)	.003 **
Phase 2	−0.08 (0.04)	−2.00 (30.19)	.055
Phase 3	−0.11 (0.05)	−2.00 (30.46)	.055
Phase 4	−0.15 (0.06)	−2.58 (26.65)	.001 *
Aggressive beahvior			
Intercept	0.68 (0.12)	5.57 (35.74)	<.000 ***
Phase 2	−0.09 (0.06)	−1.56 (31.42)	.129
Phase 3	−0.25 (0.09)	−2.64 (30.91)	.013 *
Phase 4	−0.30 (0.12)	−2.44 (27.74)	.021 *
Substance use			
Intercept	0.42 (0.14)	3.01 (35.07)	.005 **
Phase 2	−0.12 (0.11)	−1.17 (33.12)	.250
Phase 3	−0.12 (0.11)	−1.06 (32.72)	.298
Phase 4	−0.18 (0.15)	−1.21 (31.52)	.237
Distress caused by trauma			
Intercept	2.23 (0.18)	12.17 (35.60)	<.000 ***
Phase 2	−0.12 (0.08)	−1.49 (30.32)	.146
Phase 3	−0.19 (0.14)	−1.35 (30.16)	.186
Phase 4	−0.56 (0.17)	−3.31 (24.08)	.003 **
Joy			
Intercept	1.89 (0.12)	15.81 (36.06)	<.000 ***
Phase 2	0.16 (0.09)	1.81 (28.63)	.082
Phase 3	0.16 (0.10)	1.52 (30.06)	.138
Phase 4	0.40 (0.14)	2.79 (27.55)	.009 **

Note: *DF* degrees of freedom, *p < .05, **p < .01; *** *p* < .001

#### Suicidal ideation

Concerning suicidal ideation, the ICC in the first model was .79. In the next step, the comparison between the model with a fixed slope and the model with a random slope revealed a better fit for the latter model (χ^2^ (9) = 444.02, *p* < .000). In the final model, there was substantial variance between subjects. Variance for suicidal ideation at baseline (phase 1) between subjects was 0.80, and ranged from 0.06 in phase 2 to 0.22 in phase 4 for patient-specific between treatment phase variance. The residual variability was 0.23 (*n*_*obs*_ = 3855, *n* = 36), however, no significant differences between the treatment phases were found (fixed effects; Table 4).

#### Self-injury

The ICC in the model without a slope was .78. The comparison between the fixed slope and random slope models indicated a better fit for the model with a random slope (χ^2^ (9) = 107.31, *p* < .000). The estimated random effects in the final model were 0.99 at phase 1 for between patient variance and ranged from 0.03 in phase 2 to 0.06 in phase 4 for patient-specific between treatment phase variance. The 0.28 residual variability indicated substantial variance between participants (*n*_*obs*_ = 3854, *n* = 37). Concerning the fixed effects, we found a significant reduction in phase 4 compared to phase 1 (*p* < .05) (Table 4).

#### Aggressive behavior

The model without a slope had an ICC of .42. Subsequently, the comparison between the fixed slope and random slope models indicated that the inclusion of a fixed slope improved the model (χ^2^ (9) = 180.98, *p* < .000). Referring to the final model, in phase 1, the between patient variance was 0.54 and ranged from 0.06 in phase 2 to 0.36 in phase 4 for patient-specific between treatment phase variance. The residual variability was 0.60 indicating substantial variance between subjects (*n*_*obs*_ = 3761, *n* = 37). Fixed effects showed a significant decline for aggressive behavior between phase 3 compared to phase 1 (*p* < .05) as well in phase 4 compared to phase 1 (*p* < .05) (Table 4).

#### Substance use

For substance use, the ICC for the random-intercept model was .55. Again, the use of a model with a random slope suited the data better than the model with a fixed slope (χ^2^ (9) = 502.37, *p* < .000). In the final model with the random intercept and random slope, substantial variance between subjects was observed. The variance for baseline (phase 1) between subjects was 0.70 and ranged from 0.35 in phase 2 to 0.69 in phase 4 for patient-specific between treatment phase variance. The residual variability was 0.32 (*n*_*obs*_ = 3826, *n* = 37), while no significant differences between the therapy phases were found (fixed effects; Table 4).

### Research question 2

We compared the mean actions per patient for each treatment phase. The repeated measure ANOVAs with a Greenhouse-Geisser correction determined that there were no significant differences between the phases in terms of mean actions per patient for self-injurious behavior (*F* [1.63, 31.04] = 1.40, *p* = .26), aggressive behavior (*F* [1.90, 37.92] = 0.73, *p* = .48) or substance use (*F* [1.28, 24.4] = 0.58, *p* = .50).

### Secondary outcomes

In order to test for differences between the treatment phases concerning distress caused by trauma as well as joy, we also conducted MLM. Concerning distress, the ICC for the random-intercept model was .57. A comparison between the fixed slope and random slope models indicated that the inclusion of a fixed slope suited the data better (χ^2^ (9) = 377.65, *p* < .000). Estimated random effects for the final model were 1.21 for between patient variance in phase 1 and ranged from 0.14 in phase 2 to 0.70 in phase 4 for patient-specific between treatment phase variance with 0.78 residual variability, indicating substantial between subject variance (*n*_*obs*_ = 3938, *n* = 37). At the level of fixed effects, we found a significant reduction in phase 4 compared to phase 1 (*p* < .001) (Table 4).

In the case of joy, the ICC for the random-intercept model was .44. Once again, the model with a random slope suited the data better than the one with a fixed slope (χ^2^ (9) = 141.64, *p* < .000). In the final model, in phase 1, the between patient variance was 0.49 and between 0.18 in phase 2 and 0.53 in phase 4 for patient-specific between treatment phase variance with 0.90 residual variability. This indicated substantial variance between subjects (*n*_*obs*_ = 3974, *n* = 37). The fixed effects showed a significant improvement in phase 4 compared to phase 1 (*p* < .001) (Table 4).

## Discussion

To address the concerns of therapists towards trauma-focused treatments with exposure elements, the aim of the current study was to analyze whether adolescents and young adults who had experienced CA show deterioration in associated problem behaviors during intensive trauma-focused therapy preceded by skill building. For this purpose, we analyzed daily diary cards from an RCT during D-CPT [14].

Our first research question focused on possible differences between the D-CPT treatment phases regarding the urge to engage in suicidal, self-injurious, aggressive or substance use behaviors. We did not find any significant increase in rates between the D-CPT treatment phases. On the contrary, there was a significant reduction for the self-injurious and aggressive behaviors; this is contrary to the therapists’ concerns that treatments with exposure-based components could worsen PTSD-associated dysfunctional behaviors [19, 22, 27]. In fact, our results concur with existing evidence for various trauma samples, indicating that different interventions with exposure elements neither cause a deterioration in symptom severity nor in associated problems [23, 31–33]. However, compared to treatment approaches such as PE, D-CPT, as a phase-based protocol, contains preceding phases of preparation to deal with the stressful experiences during trauma-focused work [15].

More precisely, the results of the MLM analyses revealed that the patients’ urges to engage in self-injury were significantly higher at the beginning (phase 1) in comparison to the end of treatment (phase 4). In the literature it is assumed that PTSD symptoms act as a mediator for the relationship between CA and self-injury [e.g., 41, 43] and that self-injury serves as a dysfunctional coping strategy to deal with burdening re-experiences [43, 44]; this may explain why the urge for self-injury decreases during treatment. Studies on adults with comorbid borderline personality disorder showed results pointing in a similar direction; a decline in the occurrence of self-injury after trauma-focused therapy was found in comparison to that at the time of study intake [45, 47]. Nevertheless, further studies are needed which allow to draw causal conclusions about the underlying mechanisms. Furthermore, we cannot rule out that the detected improvements developed due to changes over time. Since there was no control group, it is not possible to causally relate the decline in the urge for self-injury to the intervention conducted.

There was also a significant reduction in the urge for aggressive behavior from phase 1 to phase 3 and, additionally, a significant reduction between phase 1 and phase 4. Again, PTSD symptoms have been suggested to mediate the relationship between trauma and aggressive behaviors [52, 55]. Nevertheless, data on this mediation hypothesis is inconclusive [52]. Specific interventions concerning the reduction of aggressive behavior in trauma patients are also poorly understood [74]. However, it has been recommended to address emotion regulation strategies with young patients in order to advise them of alternative approaches to regulate their emotions [52]. This is in line with findings from adults, indicating that the ability to regulate emotions accounts for the association between PTSD and aggression. [56]. Since training in emotion regulation is an integral part of D-CPT [15], this may have contributed to the detected improvements. Again, due to the lack of a control group, it remains unclear whether D-CPT has a casual effect on the decline of aggressive behaviors. Broader empirical evidence is needed before drawing definitive conclusions.

Our second research question focused on differences between the D-CPT therapy phases concerning actions of self-injurious, aggressive or substance use behaviors. Repeated measure ANOVAs found no significant differences between the treatment phases. Since phase 3 includes trauma exposure [15], deterioration in behaviors should have been (at least) evident in this phase. These results, therefore, provide further hints that the therapists’ concerns towards psychotherapy with exposure elements cannot be confirmed in the context of phase-based D-CPT treatment.

In addition to dysfunctional behaviors, we also looked at differences between treatment phases in terms of self-reported joy and distress caused by trauma. Encouragingly, there were significant improvements for both variables (decline in distress, increase in joy) from phase 1 to phase 4. This is a further indication that treatment with exposure elements, contrary to the reported concerns [19, 22, 27], improves the patient’s well-being.

### Strengths of the study

To the best of our knowledge, this is the first study that has analyzed the course of dysfunctional behaviors during intensive trauma-focused treatment in adolescent patients with abuse- related PTSD. It has, therefore, contributed to the availability of more data on young patients and the inclusion of individuals with CA, as well as comorbidities, who would otherwise be often excluded from trials due to their severe psychopathology [15, 62]. Hence, these results also counter beliefs that exposure-based therapies would be inappropriate for cases with comorbid conditions [19, 22] or multiple traumatization [22]. Another advantage of this study is that the daily assessments through diary entries provided multiple long-term data. The repeated measures helped to describe the dynamics of the dysfunctional behaviors and enabled its analysis at a within-patient level [66].

### Limitations and further research

The results are constrained by several factors. Firstly, there are methodological limitations. Although daily assessment is ecologically valid in reducing self-report bias [65], we did not use any secondary instruments for the dysfunctional behaviors examined. The additional administration of more objective measures (e.g., clinical interviews) would increase the validity in future trials. Even though the RCT during D-CPT [14] had one of the largest samples in the field of adolescent PTSD patients, the sample size of the present analysis was still small. Despite this, to model within-person dynamics, at least five observations [75] per person are needed; this requirement was fulfilled in this study. Nonetheless, there exists an ongoing debate concerning sufficient cluster sizes in this type of analysis. However, there is a clear consensus that small sample sizes increase several problems for the estimated effects [76].

For economic reasons, the diary cards were applied in the paper-pencil format. This format can favor the absence of individual entries, resulting in weeks where entries are complete contrasting weeks where there are missing entries. Furthermore, the reliability of the data is limited, as we cannot guarantee that the participants actually rated their dysfunctional behaviors on a daily basis. It is also possible that some patients answered the diary cards retrospectively just before the next treatment session began. Thus, further research should use ecological momentary assessments (e.g., smartphone-based) to foster compliance in patients [65, 77] and, thus, reduce the probability of missing data in the diary entries and increase its reliability.

Secondly, since D-CPT is designed as a phased-based protocol that incorporates emotion regulation training as a step prior to intensive CPT [15], the techniques taught may have kept the dysfunctional behaviors from increasing or, in some cases, lead to a reduction of dysfunctional behavior prior to starting the treatment phase 3 in which exposure was used. Therefore, one cannot conclude general statements about exposure-based approaches without the preceding emotion-regulation training from our data. Further research should, therefore, focus on comparing phase-based approaches such as D-CPT versus approaches with exposure elements only. The self-monitoring process may also have changed the frequency of the examined dysfunctional behaviors. Although reactivity in daily assessments is rather scarce, some studies have found such effects [e.g., 78], which can only be detected in the cases of large sample sizes and an appropriate control group.

Thirdly, descriptive statistics show that dysfunctional behaviors were infrequent in this sample as the mean values all tended to fall within the range of the scale minimum. This may have restricted the investigated variance and, thus, only allows for conclusions to be drawn on the present trial without any statements about more symptomatic patients. Our exclusion criteria may also have contributed towards this since no present substance dependence, life-threatening self-injury or suicidality were allowed as the treatment would, otherwise, not have been feasible in an outpatient setting. Another contributing factor may have been that the diary cards were also used for therapeutic purposes. The participants were aware that their answers would be read and discussed during treatment. Consequently, we cannot rule out that the patients indicated a lower urge to engage in dysfunctional behaviors in order to please the therapist.

Fourthly, we are unable to estimate the effect of therapist characteristics on our results. On the one hand, there is recent evidence from Trauma-Focused Cognitive Behavioral Therapy indicating that general therapist characteristics (e.g., age, clinical expertise, theoretical background) do not have an effect on treatment outcome in children and adolescents [79]. On the other hand, more specific therapist characteristics such as the extent of negative beliefs towards trauma exposure seem to be connected with the suboptimal delivery of exposure elements [20, 24]. We assume that the therapists in our study had mostly positive beliefs about the intervention and its exposure elements, which, in turn, had a positive effect on the treatment delivery. Moreover, different therapists may have placed different emphasis on the patient’s compliance with filling in the diary cards. Therefore, future studies should assess therapist’s beliefs towards exposure-based treatments and filling in diary cards as well as more general therapist characteristics to examine their effect on patients’ dysfunctional behaviors during treatment.

### Clinical implications

The presented results indicate that the negative beliefs of some therapists need to be revised. There are therapist trainings that lead to a reduction of these negative apprehensions, improving the practitioner’s motivation for applying treatment with exposure elements [e.g., 80, 81]. Another promising approach in the therapists’ continuing education is regular supervision [82]. Targeted supervision addressing negative beliefs should have a positive impact on the dissemination of trauma-focused therapy services among adolescents and young adults with PTSD [83].

## Conclusions

This study is the first to analyze dysfunctional behaviors during D-CPT and one of the few on adolescents and young adults with abuse-related PTSD. We were able to draw on the daily diary data from the RCT by Rosner et al. [14] and, thus, additionally map the individual patient’s dynamics. We found no significant differences between therapy phases with respect to the studied dysfunctional behaviors and even observed partial improvements. However, further studies with extended measurement methods in terms of ecological momentary assessment, a control group and larger samples sizes are needed to confirm these preliminary results. Nevertheless, we hope that these findings contribute to enhance routine care through the dissemination of evidence-based approaches such as D-CPT.

## Data Availability

The datasets and materials generated during and/or analyzed during the current study are available from the corresponding author on reasonable request.

## Abbreviations

ANOVA: Analysis of variance
CA: Child sexual and/or physical abuse
CAPS-CA: Clinician-Administered PTSD Scale for Children and Adolescents
CPT: Cognitive Processing Therapy
CPTSD: Complex posttraumatic stress disorder
D-CPT: Developmentally adapted Cognitive Processing Therapy
DF: Degrees of freedom
ICC: Interclass correlation
M: Mean
N: Number of participants
N_diary_: Number of diary cards
N_obs._: Number of observations in terms of diary card entries
MLM: Multilevel modeling
PE: Prolonged exposure
PTSD: Posttraumatic stress disorder
RCT: Randomized controlled trial
SD: Standard deviation
UCLA-PTSD-RI: University of California at Los Angeles PTSD Reaction Index for DSM-IV (Revision 1)
WL/TA: Wait-list condition with treatment advice
